# Prevalence and Diagnostic Determinants of Hepatitis B Infection Among Saudi Adults: Implications for Targeted Screening and Early Detection

**DOI:** 10.3390/diagnostics15233050

**Published:** 2025-11-29

**Authors:** Mohammad A. Jareebi, Ali A. Awam, Dhiyaa A. H. Otayf, Saja A. Almraysi, Israa H. Alqamaryat, Amaal A. Alghamdi, Majed A. Ryani, Ahmed A. Bahri, Abdulwahab A. Aqeeli, Jamaludeen A. Othman, Adhari A. Alselmi, Farjah H. Algahtani, Hani A. Alghamdi, Ghazi I. Al Jowf, Aisha H. Majrashi

**Affiliations:** 1 Department of Family and Community Medicine, Jazan University, Jazan 45142, Saudi Arabia; majedryani@gmail.com (M.A.R.); dr.bahri2010@gmail.com (A.A.B.); aaqeeli@jazanu.edu.sa (A.A.A.); 2 Department of Preventive Medicine, Ministry of Health, Jubail 31951, Saudi Arabia; ali88a2015@gmail.com (A.A.A.); israahassan799@gmail.com (I.H.A.);; 3 Faculty of Medicine, Jazan University, Jazan 45142, Saudi Arabia; dhiyaaot@gmail.com (D.A.H.O.); sajaalasiri1@gmail.com (S.A.A.); 4 Jazan University Hospital, Jazan 45142, Saudi Arabia; dr.zilay@gmail.com; 5 Clinical Sciences Department, MBBS Program Fakeeh College for Medical Sciences, Jeddah 21461, Saudi Arabia; adhari.alselmi@hotmail.com; 6 Oncology Center, Epidemiology and Public Health Research Chair, Faculty of Medicine, King Saud University/King Saud Medical City, Riyadh 12373, Saudi Arabia; falgahtani@ksa.edu.sa; 7 Department of Family and Community Medicine, College of Medicine, King Saud University, Riyadh 12373, Saudi Arabia; halhajalah@ksu.edu.sa; 8 Department of Public Health, College of Applied Medical Sciences, University Medical Clinics Complex, King Faisal University, Al Hofuf 37912, Saudi Arabia; galjowf@kfu.edu.sa; 9 Jazan Health Cluster, Southern Sector, Primary Health Care Center, Ramadah, Jazan 45142, Saudi Arabia; aishamajrashi4@gmail.com

**Keywords:** hepatitis B virus, diagnostic screening, risk factors, diabetes mellitus, Saudi Arabia, adult vaccination, public health

## Abstract

**Background/Objectives:** Hepatitis B virus (HBV) remains a significant diagnostic and public health challenge worldwide. Despite widespread vaccination, underdiagnosis persists among adults in Saudi Arabia. This study estimated HBV prevalence and identified sociodemographic, clinical, and behavioral predictors relevant to improving targeted diagnostic screening. **Methods**: A cross-sectional study of 1196 Saudi adults aged ≥18 years was conducted between September 2024 and February 2025 using a structured questionnaire. Data on demographics, clinical history, and behavioral exposures were analyzed using chi-square tests and multivariate logistic regression to identify independent determinants of HBV infection. **Results**: The study included 1196 adults (60.0% female, mean age 31 ± 12 years). HBV prevalence was 2.0% (95% CI: 1.3–3.0%). Independent predictors included divorced/widowed marital status (OR = 3.99, *p* = 0.023), diabetes mellitus (OR = 3.59, *p* = 0.039), family history of HBV (OR = 2.55, *p* < 0.001), and massage exposure (OR = 3.99, *p* = 0.025). No significant associations were found with gender, education, or transfusion history. **Conclusions**: HBV infection persists among high-risk Saudi adults despite immunization success. Integrating HBV testing into diabetes care, premarital and household screening, and regulation of personal care services may enhance early diagnosis and advance national elimination goals.

## 1. Introduction

Hepatitis B virus (HBV) infection represents a major global public health challenge, affecting approximately 296 million people worldwide [1,2] and causing an estimated 820,000 deaths annually, primarily from complications such as liver cirrhosis [3] and hepatocellular carcinoma [4]. HBV is transmitted through contact with infected blood and body fluids via several routes, including mother-to-child transmission during childbirth, unprotected sexual contact, exposure to contaminated blood through shared needles or unsafe transfusions, and use of unsterilized medical or dental instruments [5]. The virus can also spread through sharing personal items such as razors or toothbrushes contaminated with blood [6] or through traditional practices such as unregulated massage involving shared instruments [7]. Furthermore, diabetes mellitus and a positive family history of HBV increase vulnerability, partly through greater exposure to healthcare interventions. Globally, HBV-related awareness and treatment remain critically low, with only a small proportion of infected individuals aware of their status and even fewer receiving therapy. HBV prevalence shows significant geographical variation, with countries in the Middle East categorized as having intermediate endemicity, with prevalence rates ranging from 2% to 8% [8]. This epidemiological pattern is primarily attributed to political instability and inadequate healthcare infrastructure, which have hindered effective vaccination campaigns in many areas. In Saudi Arabia, HBV prevalence, which was estimated at 3.2% between 1965 and 2013, declined to 1.3% by 2021 [9]. This substantial reduction is primarily attributable to the introduction of a national HBV vaccination program in the 1990s, coupled with robust public health awareness campaigns [9]. Despite notable advances in prevention efforts, including neonatal vaccination coverage and implementation of safe blood and injection practices, progress toward hepatitis elimination goals remains insufficient. Early detection of HBV is crucial to prevent disease progression and reduce transmission, particularly in adult populations who may be asymptomatic yet at risk of severe complications. Critically, early diagnosis and linkage to treatment remain limited: only 7% of HBV cases are detected and merely 4% receive treatment in Saudi Arabia, far below WHO targets of 90% diagnosis and 80% treatment [10]. In addition to these health system gaps, Saudi Arabian cultural norms, such as extended family living arrangements, shared grooming routines, and traditional practices like wet cupping, significantly increase the risk of HBV exposure, particularly when hygiene standards are inadequate [11]. Addressing these determinants is imperative to achieve the World Health Organization’s ambitious hepatitis elimination targets by 2030, which include a 90% reduction in HBV incidence and a 65% reduction in HBV-related mortality [12]. Strengthening early detection through targeted screening is therefore essential. Therefore, this study aims to determine the prevalence of HBV among adults in Saudi Arabia and to identify key demographic and clinical determinants to inform diagnostic screening strategies and national elimination policies. It is important to note that this study relies on self-reported HBV status rather than serological confirmation. Acknowledging this constraint from the outset frames the interpretation of prevalence estimates and associated factors. The findings are expected to inform evidence-based public health strategies and targeted interventions to improve early detection, treatment uptake, and overall management of HBV in the adult population of Saudi Arabia.

## 2. Materials and Methods

### 2.1. Study Design and Setting

A cross-sectional analytical study was conducted across Saudi Arabia over a year, from September 2024 to September 2025. Eligible participants included Saudi nationals aged 18 years and older who provided informed consent and completed the study questionnaire in its entirety. Exclusion criteria comprised non-Saudi residents, individuals under 18 years of age, those unable or unwilling to provide informed consent, and participants with incomplete questionnaire responses.

### 2.2. Sampling and Sample Size Calculation

**Figure 1:**
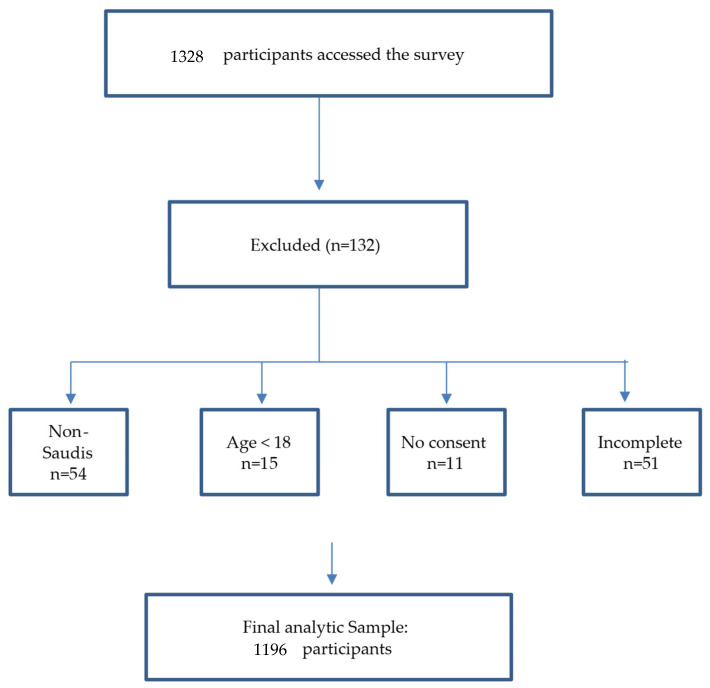
Participant flowchart illustrating inclusion and exclusion criteria among Saudi adults.

Participants were recruited using a convenience sampling technique through online platforms and community health centers. Approximately 80% of participants were recruited online via social media platforms, while the remaining 20% were recruited through community health centers. Participants were drawn from multiple regions across Saudi Arabia to enhance representativeness. According to the General Authority for Statistics (GASTAT), Saudi Arabia’s total population in 2025 is approximately 37.7 million, of which 64.8% are adults aged 20–64 years, equating to approximately 24.2 million adults [13]. The target population for this study was conservatively estimated at approximately 20 million Saudi adults aged 18 years and older. The minimum required sample size was calculated using the single population proportion formula for a 95% confidence level, an estimated HBV prevalence of 2% (based on recent national data [9] and accounting for undiagnosed cases), and a margin of error of 1.5%:

$$n=\frac{Z^{2}\cdot p\cdot (1-p)}{E^{2}}$$

where *Z* = 1.96 (standard score for 95% CI), *p* = anticipated prevalence, and *E* = margin of error. This yielded an initial sample size of 335 participants. Accounting for 20% non-response and the need for multivariate analysis, the target was increased to 1000 participants. A total of 1328 individuals accessed the survey. Of these, 132 responses were excluded (non-Saudi nationals = 54, age <18 years = 15, no consent = 11, incomplete responses = 51). The final analytic sample included 1196 eligible participants (Figure 1). Ultimately, 1196 adults were enrolled, exceeding requirements and enhancing statistical power.

### 2.3. Data Collection

Data were collected using a structured, pre-tested questionnaire available in both Arabic and English to ensure accessibility. The questionnaire was developed based on previously published HBV epidemiological studies and was reviewed by subject-matter experts for content validity. It was then pilot tested on a small group of 30 participants to ensure clarity and reliability, with minor modifications, rewording sociodemographic questions, simplifying the language of clinical history items, and adding brief explanatory notes to the ‘massage exposure’ section, incorporated before full deployment. The survey was disseminated online via Google Forms and shared on social media platforms such as WhatsApp, X, and Telegram to reach a diverse demographic of Saudi adults.

The questionnaire gathered comprehensive data across several domains:

**Sociodemographic characteristics**: age, sex, residence (urban/rural), education level, marital status, polygamy status, and monthly income.

**Health history**: physician-diagnosed diabetes mellitus, hypertension, and history of surgical procedures.

**Behavioral exposures**: smoking status (never/ex-smoker/current), physical activity levels, history of blood transfusion or donation, and exposure to massage services.

**Travel history**: international travel destinations (regular traveler).

**Family history**: family history of hepatitis B infection.

**HBV infection status** was self-reported by participants based on prior medical diagnosis. Participants were asked whether they had ever been diagnosed with Hepatitis B by a healthcare provider following standard diagnostic procedures, such as routine screening during hospital visits, pre-employment testing, premarital screening, or clinical evaluation prompted by symptoms. These diagnoses are typically confirmed through serological testing (e.g., HBsAg), which participants reported as part of their medical history. No additional serological testing was performed for this study. The primary outcome was binary: diagnosed with HBV versus not diagnosed with HBV.

### 2.4. Data Analysis

Following data collection, raw data were exported to Excel for error checking, validation, and assessment of missing values. Subsequent statistical analyses were performed using R software (version 4.2.3; R Foundation for Statistical Computing, Vienna, Austria). Descriptive statistics (frequencies, percentages, mean ± SD) were used to summarize participant characteristics. Data normality was assessed using the Shapiro–Wilk test; however, as the study outcomes were categorical and analyzed using chi-square tests and multivariable logistic regression, normality assumptions were not required for the primary analyses. Chi-square tests (χ^2^) were utilized to examine univariate associations between categorical variables and HBV status. Variables with *p* < 0.05 in univariate analyses as well as variables with established biological plausibility or identified as potential confounders (such as age and gender) were included in a multivariate logistic regression model to identify independent predictors of HBV infection. Odds ratios (ORs) with 95% confidence intervals (CIs) were calculated to assess the strength of associations. Statistical significance was set at *p* < 0.05 (two-tailed).

### 2.5. Ethical Considerations

Ethical approval for this study was obtained from the Standing Committee for Sabbatical Leaves, Publication and Research Ethics at Jazan University (REC-46/02/1167, dated 1 September 2024). All study procedures complied with the principles outlined in the Declaration of Helsinki. Written informed consent was obtained from all participants before inclusion. To ensure data protection and confidentiality, online responses were collected through Google Forms and stored in password-protected, encrypted files. No identifying information was recorded. All data were anonymized before analysis to prevent participant identification.

### 2.6. Use of Generative Artificial Intelligence

Generative AI was used solely for language editing. Study design, data, analysis, and interpretation were entirely conducted by the authors.

## 3. Results

### 3.1. Sociodemographic Characteristics

The study enrolled 1196 Saudi adults, comprising 718 females (60.0%) and 478 males (40.0%), with a mean age of 31 ± 12 years. Most participants resided in urban areas (89.0%, n = 1065), while 11.0% (n = 131) lived in rural settings. Regarding marital status, more than half of participants were single (53.0%, n = 634), 43.0% (n = 514) were married, and 4.0% (n = 48) were divorced or widowed. Polygamous marriages were uncommon, representing only 6.0% (n = 72) of the sample. Educational attainment was notably high, with 71.0% (n = 849) holding university degrees, 22.0% (n = 263) having completed high school or lower education, and 7.0% (n = 84) possessing postgraduate qualifications. Monthly income distribution showed considerable variation: 30.0% (n = 359) earned more than 15,000 SAR, 27.0% (n = 323) earned 10,000–14,999 SAR, 19.0% (n = 227) earned 5000–9999 SAR, and 24.0% (n = 287) earned less than 5000 SAR (Table 1).

### 3.2. Health Status and Behavioral Characteristics

Chronic diseases were relatively uncommon in the study population. Diabetes mellitus was reported by 6.0% (n = 72) of participants, while hypertension was present in 7.0% (n = 84). A history of surgical procedures was reported by 34.0% of participants (n = 407). Regarding lifestyle behaviors, the majority of participants (84.0%, n = 1005) had never smoked, while 9.0% (n = 107) were current smokers and 7.0% (n = 84) were ex-smokers. More than half of the participants (56.0%, n = 670) engaged in regular physical activity. A small proportion (3.0%, n = 36) reported having visited an addiction clinic (Table 2).

### 3.3. HBV Prevalence and Transmission Risk Factors

The overall prevalence of HBV infection in the study population was 2.0% (n = 24). Given the small number of HBV-positive cases, caution is warranted when interpreting subgroup percentages, as small denominators may exaggerate apparent differences. Several potential HBV transmission risk factors were assessed. A history of blood transfusion or donation was reported by 12.0% (n = 143) of participants, while 10.0% (n = 120) reported exposure to massage services. Notably, 8.0% (n = 96) of participants reported a family history of hepatitis B infection. Regarding international travel history, 45.0% (n = 538) had never traveled outside Saudi Arabia. Among those who had traveled, 24.0% (n = 287) had visited Arab Gulf countries, 11.0% (n = 131) Europe, 9.0% (n = 108) Asia, 7.0% (n = 84) other Middle Eastern countries, 3.0% (n = 36) Africa, and 1.0% (n = 12) the Americas (Table 3).

### 3.4. Univariate Analysis of Factors Associated with HBV Infection

Chi-square analyses revealed statistically significant associations between HBV infection and several key sociodemographic and health-related factors (Table 4). Marital status showed a significant association with HBV infection (*p* = 0.011), with HBV prevalence substantially higher among divorced or widowed individuals (8.3%, n = 4/48) compared to married participants (2.3%, n = 12/514) and single individuals (1.3%, n = 8/634). Diabetes mellitus demonstrated a strong association with HBV infection (*p* < 0.001), with the prevalence among diabetic participants at 8.3% (n = 6/72), significantly higher than the 1.6% (n = 18/1124) observed among non-diabetic individuals. Similarly, massage exposure was significantly associated with HBV infection (*p* < 0.001), with HBV prevalence of 6.7% (n = 8/120) among participants with a history of massage services compared to only 1.5% (n = 16/1076) among those without such exposure. Family history of hepatitis B showed the strongest univariate association (*p* < 0.001), with participants reporting a positive family history having an HBV prevalence of 12.5% (n = 12/96), markedly higher than the 1.1% (n = 12/1100) observed among those without a family history. In contrast, no statistically significant associations were observed between HBV infection and gender (*p* = 0.248), age (*p* = 0.162), residence (*p* = 0.297), education level (*p* = 0.419), monthly income (*p* = 0.582), polygamy status (*p* = 0.285), smoking status (*p* = 0.451), physical activity (*p* = 0.534), blood transfusion history (*p* = 0.811), surgical history (*p* = 0.348), hypertension (*p* = 0.933), addiction clinic visits (*p* = 0.204), or international travel destinations (*p* values ranging from 0.128 to 0.881 across all regions).

### 3.5. Multivariate Predictors of HBV Infection

**Figure 2:**
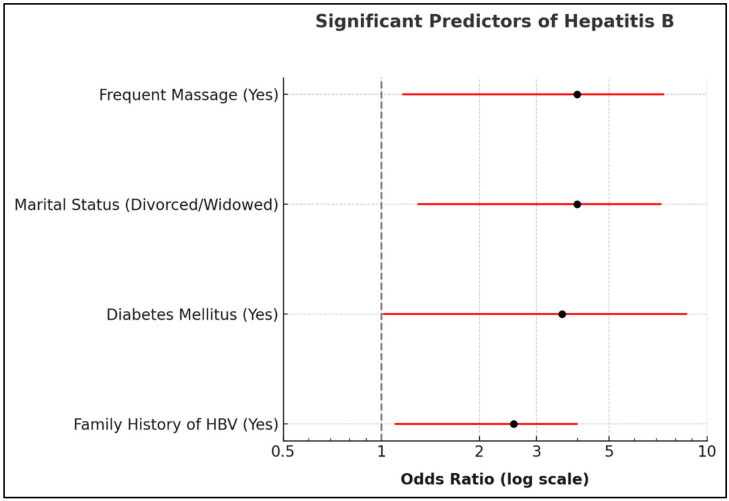
Significant Predictors of Hepatitis B. Forest plot of odds ratios (OR) with 95% confidence intervals for significant predictors. The *x*-axis is on a logarithmic scale, ensuring symmetric interpretation of risk (OR > 1) and protective (OR < 1) factors around the null value (OR = 1).

Multiple logistic regression analysis identified four independent predictors of HBV infection after adjusting for potential confounders, including age, gender, and other relevant covariates (Table 5 and Figure 2). Divorced or widowed marital status emerged as a significant predictor, with affected individuals demonstrating nearly four times higher odds of HBV infection compared to single individuals (OR = 3.99; 95% CI: 1.29–12.35; *p* = 0.023). Diabetes mellitus was independently associated with HBV infection, with diabetic participants exhibiting 3.59 times higher odds of infection compared to non-diabetic individuals (OR = 3.59; 95% CI: 1.01–12.78; *p* = 0.039). Massage exposure demonstrated a strong independent association with HBV infection, conferring 3.99 times higher odds of infection (OR = 3.99; 95% CI: 1.16–13.73; *p* = 0.025). Family history of hepatitis B emerged as the most consistent predictor across the model, more than doubling the odds of HBV infection (OR = 2.55; 95% CI: 1.10–5.91; *p* < 0.001). Other variables examined in the multivariate model, including gender, age, residence, education level, income, polygamy status, smoking status, physical activity levels, surgical history, blood transfusion history, hypertension, addiction clinic visits, and travel history, did not demonstrate statistically significant independent associations with HBV infection after adjustment for confounders (all *p* > 0.05). The logistic regression model demonstrated adequate predictive performance, with a Tjur’s pseudo-R^2^ of 0.281, indicating that the model explained approximately 28.1% of the variance in HBV diagnosis.

## 4. Discussion

### 4.1. Principal Findings

This cross-sectional study investigated the prevalence and determinants of HBV infection among 1196 Saudi adults, identifying both sociodemographic and behavioral risk factors that significantly influence infection patterns. The overall HBV prevalence of 2.0% reflects the progress made through the national vaccination program in Saudi Arabia, but it also underscores the persistence of infection among specific subgroups. The independent predictors included divorced or widowed marital status, diabetes mellitus, family history of HBV, and exposure to massage. These predictors highlight associations with HBV infection, reflecting potential biological and sociocultural factors influencing transmission. From a diagnostic perspective, these findings underscore the persistence of infection among underdiagnosed adult subgroups. Strengthening detection strategies that prioritize individuals with identified risk profiles—such as diabetic adults, those with positive family histories, and divorced or widowed individuals—could substantially improve early case identification and linkage to care within the national health system.

### 4.2. HBV Prevalence in Context: Comparison with National and International Data

The prevalence observed in this study is broadly consistent with previous national data, though variations exist. A large Saudi premarital screening study reported in 2012 an HBV prevalence of 1.3% [14], while another study conducted in 2017 documented 1.7% HBV prevalence in Riyadh adults [15]. By comparison, our prevalence is slightly higher, which may reflect differences in regional exposure patterns or the inclusion of high-risk groups in our sample. Contrastingly, much higher prevalence has been reported in Egypt (3.2%) in 2022 [16] and Pakistan (5–7%) in 2022 [17], while rates in China remain as high as 6–8% in 2020 [18]. The substantially lower prevalence among vaccinated Saudi adolescents, where it approaches zero [19], demonstrates the long-term effectiveness of the universal vaccination policy introduced in 1989. However, the persistence of infection among adults may reflect ongoing gaps in case finding, particularly among unvaccinated cohorts. Because only 24 participants in our sample were HBV-positive, subgroup comparisons should be interpreted with caution, and observed percentage differences may be influenced by small denominators.

### 4.3. Diabetes Mellitus as a Risk Factor: Biological and Clinical Implications

Diabetes mellitus was an independent predictor, with affected individuals showing 3.6-fold higher odds of HBV infection. This association is strongly supported in international literature. A U.S. study demonstrated a nearly 1.9-fold increased risk of acute HBV in diabetic adults [20], while studies found a 1.5-fold increase in Chinese populations [21]. CDC reports suggest that adults with diabetes have a 60% higher prevalence of HBV markers and nearly double the odds of acute infection compared to non-diabetic adults [22]. Several biological and epidemiological mechanisms explain this association [23]. First, diabetic individuals undergo more frequent medical interventions, including blood glucose monitoring, insulin injections, and hospitalizations, which increase opportunities for blood-borne exposure in clinical settings [24]. Second, diabetes is associated with immune dysfunction, which may impair the ability to clear HBV infection. Evidence also suggests a dose–response effect, with longer diabetes duration and insulin dependence further elevating risk [25]. Although our study did not measure disease duration, these findings suggest that targeted vaccination and screening for diabetic adults may be beneficial and warrant further investigation in Saudi Arabia.

### 4.4. Marital Status and HBV: The Role of Cultural Context and Social Vulnerability

Marital status emerged as a strong predictor, with divorced and widowed participants exhibiting nearly fourfold increased odds of HBV infection compared to single individuals. These findings are in line with a study in Iran that also documented elevated infection risk among divorced or widowed adults [26]. Regional research suggests that marital transition is a proxy for increased exposure to unprotected sexual contact and reduced partner screening, thereby increasing susceptibility to infection [27]. However, contrasting findings from Jordan reported no significant association between marital status and HBV [28], possibly due to cultural differences in reporting sexual behavior and stigma-related underdiagnosis. In the Saudi context, cultural norms and stigma are central to understanding this pattern [7,29]. Divorced and widowed individuals, particularly women, may face reduced access to healthcare and screening due to financial dependency, restricted mobility, and social stigma surrounding sexually transmitted infections. Fear of discrimination may discourage them from seeking HBV testing or vaccination, leading to underreporting and delayed detection. These results suggest that including marital history in screening programs could help identify socially vulnerable groups, which may inform future health promotion strategies. Recognizing these marital-status-related disparities is important for HBV management, as targeted counseling, vaccination outreach, and routine screening of divorced and widowed individuals could support earlier diagnosis and reduce undetected transmission.

### 4.5. Family History and Intrafamilial Transmission Dynamics

Participants with a family history of HBV had 2.6 times higher odds of infection, consistent with intrafamilial transmission dynamics. Vertical transmission from mother to child at birth is a well-established pathway, especially in the absence of timely immunoprophylaxis [30]. Horizontal transmission within households also plays a significant role, with exposure to shared razors, toothbrushes, or minor wounds acting as potential transmission routes. Studies in China and Taiwan report intrafamilial prevalence of 10–25% among household contacts [31]. In Saudi Arabia, extended family living arrangements may amplify these risks and may support family-based screening and vaccination strategies that are essential. These findings underscore the need for policy measures promoting routine family-based screening, targeted vaccination of household contacts, and educational campaigns to reduce intrafamilial transmission of HBV.

### 4.6. Massage Exposure: An Overlooked Transmission Route

Massage exposure demonstrated fourfold increased HBV odds, biologically plausible given HBV stability in dried blood and transmission via microscopic percutaneous exposures [32,33]. Importantly, this association may be partially confounded by travel history. While personal care establishments in Saudi Arabia are closely monitored under health authority regulations and Saudi Vision 2030 infection prevention standards, participants may have accessed massage services during international travel to countries with less stringent infection control. Although univariate analysis showed no significant associations with specific travel destinations, the interaction between travel and massage exposure warrants further investigation. These findings underscore the potential value of traveler education regarding infection risks associated with personal care services abroad, alongside continued domestic regulatory oversight.

### 4.7. Saudi Vision 2030 and Progress Toward HBV Elimination

Saudi Vision 2030 has significantly advanced HBV control through multiple initiatives. First, expanded universal health coverage has improved access to vaccination and screening services, integrated into primary care, maternal-child health programs, and premarital screening [9]. Second, enhanced infection prevention standards across healthcare facilities have reduced iatrogenic transmission [10]. Third, stricter licensing and inspection requirements for personal care services ensure sterilization compliance [11]. Fourth, nationwide health education campaigns have addressed transmission routes and vaccination importance, particularly targeting high-risk populations. These initiatives have facilitated Saudi Arabia’s epidemiological transition from intermediate to low HBV endemicity. Sustained efforts in adult screening, stigma reduction, and equitable healthcare access for high-risk and marginalized groups remain critical in achieving HBV elimination by 2030.

### 4.8. Public Health Implications

The implications of this study for public health are significant. First, targeted screening for high-risk groups, such as divorced or widowed adults, diabetics, and individuals with familial histories, may be beneficial and warrants further evaluation. Second, enhancing infection control measures in personal service settings, including massage, barber shops, and beauty salons, could help reduce transmission risks, complemented by public awareness efforts to inform travelers about potential infection exposures abroad. Moreover, diagnostic coverage for HBV in Saudi Arabia remains limited, and most adult infections are identified incidentally; integrating HBV testing into chronic disease clinics, family programs, and premarital screening may support earlier case detection and linkage to care. Third, community-based awareness campaign initiatives could also contribute to reducing stigma and promoting HBV testing, especially among marginalized populations. Fourth, strengthening adult vaccination efforts alongside robust family-based screening may help address the gaps left by childhood immunization programs. Finally, evaluating the potential cost-effectiveness of expanding vaccination coverage and incorporating molecular epidemiology could provide useful insights for understanding HBV transmission dynamics and guiding future strategies aligned with Saudi Arabia’s Vision 2030 health objectives.

### 4.9. Strengths and Limitations

This study has notable strengths. The large sample size (n = 1196) exceeds the calculated minimum requirement, providing adequate statistical power and precise prevalence estimates for subgroup analyses. The study systematically evaluated multiple risk factors and exposures across sociodemographic, clinical, and behavioral domains, enabling identification of both established and novel predictors. A comprehensive statistical investigation employed both univariate analysis to identify potential associations and multivariate logistic regression to determine independent predictors while controlling for confounders, strengthening the internal validity of findings.

However, several limitations warrant consideration. Because HBV status was self-reported, reliance on self-reported HBV status without serological confirmation may have led to misclassification, potentially affecting both prevalence estimates and the strength of observed associations. The cross-sectional design precludes establishing causality or temporal relationships. Convenience sampling with predominant online recruitment may have introduced selection bias, overrepresenting educated urban populations while underrepresenting vulnerable groups, limiting generalizability. Self-reported data for exposures such as marital history, diabetes status, family history of HBV, blood transfusion/donation, and massage exposure introduce recall and social desirability bias, which may further influence the accuracy of exposure reporting and the strength of observed associations. The small number of HBV-positive cases (n = 24) resulted in wide confidence intervals, limiting precision. Additionally, small denominators in certain subgroups may exaggerate apparent differences, warranting caution when interpreting subgroup percentages. Nevertheless, this limitation itself underscores the diagnostic gap in HBV surveillance—many adults remain undiagnosed until late-stage disease, highlighting the potential value of risk-based screening and diagnostic outreach. Unmeasured confounders, including vaccination history, diabetes duration, healthcare access patterns, massage establishment types (domestic versus abroad), and timing of exposures relative to travel, were not captured. Despite these limitations, the findings provide valuable insights into HBV epidemiology and identify high-risk subgroups that may benefit from targeted interventions, which could be considered in alignment with the Saudi Vision 2030 framework. These findings should be interpreted cautiously and viewed as preliminary evidence that warrants further research using confirmed clinical data to validate prevalence estimates and explore potential causal relationships.

## 5. Conclusions

This study suggests that hepatitis B virus (HBV) infection persists among Saudi adults, with a prevalence of 2.0% despite longstanding childhood vaccination programs. Marital status, diabetes mellitus, family history of HBV, and massage exposure emerged as potential determinants that may be associated with an increased risk of HBV infection. These findings suggest that targeted screening, integration of HBV testing into diabetes care, support for family-based screening, and attention to infection-control practices in personal care settings may help in the identification of undiagnosed cases and improve early linkage to care. Future research, including studies with serologically confirmed HBV status, is warranted to validate these patterns, clarify causal relationships, and inform evidence-based strategies for HBV prevention and control in Saudi Arabia.

## Figures and Tables

**Table 1:** Sociodemographic Characteristics of Study Participants (n = 1196).

Variable	Mean ± SD	
**Age (years)**	31 ± 12	
**Variable**	Category	n (%)
**Gender**	Female	718 (60.0)
	Male	478 (40.0)
**Residence**	Urban	1065 (89.0)
	Rural	131 (11.0)
**Marital Status**	Single	634 (53.0)
	Married	514 (43.0)
	Divorced/Widowed	48 (4.0)
**Polygamy**	Yes	72 (6.0)
	No	1124 (94.0)
**Education Level**	High school or lower	263 (22.0)
	University degree	849 (71.0)
	Postgraduate	84 (7.0)
**Monthly Income (SAR)**	<5000	287 (24.0)
	5000–9999	227 (19.0)
	10,000–14,999	323 (27.0)
	>15,000	359 (30.0)

**Table 2:** Health Status and Lifestyle Characteristics (n = 1196).

Characteristic	Category	n (%)
**Diabetes Mellitus**	Yes	72 (6.0)
	No	1124 (94.0)
**Hypertension**	Yes	84 (7.0)
	No	1112 (93.0)
**Surgery History**	Yes	407 (34.0)
	No	789 (66.0)
**Smoking Status**	Never smoked	1005 (84.0)
	Ex-smoker	84 (7.0)
	Current smoker	107 (9.0)
**Physical Activity**	Regular	670 (56.0)
	Less regular/None	526 (44.0)
**Addiction Clinic Visit**	Yes	36 (3.0)
	No	1160 (97.0)

**Table 3:** Hepatitis B Transmission Risk Factors (n = 1196).

Characteristic	Category	n (%)
**Blood Transfusion/Donation**	Yes	143 (12.0)
	No	1053 (88.0)
**Massage Exposure**	Yes	120 (10.0)
	No	1076 (90.0)
**Family History of HBV**	Yes	96 (8.0)
	No	1100 (92.0)
**Travel History**	Never traveled outside KSA	538 (45.0)
	Arab Gulf countries	287 (24.0)
	Europe	131 (11.0)
	Asia	108 (9.0)
	Middle Eastern countries	84 (7.0)
	Africa	36 (3.0)
	Americas	12 (1.0)

**Table 4:** Univariate Analysis—Factors Associated with Hepatitis B Infection.

Variable	No HBV	HBV	*p*-Value *
**Marital Status**			0.011
Single, n (%)	634 (53.0%)	8 (1.3%)	
Married, n (%)	514 (43.0%)	12 (2.3%)	
Divorced/Widowed, n (%)	48 (4.0%)	4 (8.3%)	
**Diabetes Mellitus**			<0.001
Yes, n (%)	72 (6.0%)	6 (8.3%)	
No, n (%)	1124 (94.0%)	18 (1.6%)	
**Massage Exposure**			<0.001
Yes, n (%)	120 (10.0%)	8 (6.7%)	
No, n (%)	1076 (90.0%)	16 (1.5%)	
**Family History of HBV**			<0.001
Yes, n (%)	96 (8.0%)	12 (12.5%)	
No, n (%)	1100 (92.0%)	12 (1.1%)	
**Gender**			0.248
Female, n (%)	718 (60.0%)	12 (1.7%)	
Male, n (%)	478 (40.0%)	12 (2.5%)	
**Residence**			0.297
Urban, n (%)	1065 (89.0%)	20 (1.9%)	
Rural, n (%)	131 (11.0%)	4 (3.1%)	
**Education Level**			0.419
High school or lower, n (%)	263 (22.0%)	7 (2.7%)	
University degree, n (%)	849 (71.0%)	15 (1.8%)	
Postgraduate, n (%)	84 (7.0%)	2 (2.4%)	
**Smoking Status**			0.451
Never smoked, n (%)	1005 (84.0%)	19 (1.9%)	
Ex-smoker, n (%)	84 (7.0%)	2 (2.4%)	
Current smoker, n (%)	107 (9.0%)	3 (2.8%)	
**Blood Transfusion/Donation**			0.811
Yes, n (%)	143 (12.0%)	3 (2.1%)	
No, n (%)	1053 (88.0%)	21 (2.0%)	
**Surgery History**			0.348
Yes, n (%)	407 (34.0%)	6 (1.5%)	
No, n (%)	789 (66.0%)	18 (2.3%)	
**Hypertension**			0.933
Yes, n (%)	84 (7.0%)	2 (2.4%)	
No, n (%)	1112 (93.0%)	22 (2.0%)	

* Chi-square test (or Fisher’s exact test where appropriate); bold *p*-values indicate statistical significance (*p* < 0.05).

**Table 5:** Multiple Logistic Regression—Independent Predictors of HBV Infection.

Predictor *	Odds Ratio (OR)	95% CI	*p*-Value
Gender (Male vs. Female)	1.71	0.58–5.02	0.329
Age (per year increase)	0.96	0.91–1.02	0.183
Residence (Urban vs. Rural)	0.51	0.15–1.73	0.297
**Marital Status—Divorced/Widowed (vs. Single)**	**3.99**	**1.29–12.35**	**0.023**
Marital Status—Married (vs. Single)	3.15	0.78–12.72	0.108
Polygamy (Yes vs. No)	0.34	0.04–2.89	0.285
Education—Postgraduate (vs. High school or lower)	0.16	0.01–2.15	0.146
Education—University (vs. High school or lower)	0.69	0.22–2.17	0.531
Income 10,000–14,999 SAR (vs. <5000)	0.52	0.12–2.26	0.369
Income 5000–9999 SAR (vs. <5000)	0.46	0.09–2.34	0.331
Income >15,000 SAR (vs. <5000)	0.72	0.18–2.88	0.633
Smoking—Ex-smoker (vs. Never)	2.33	0.41–13.25	0.294
Smoking—Current (vs. Never)	1.51	0.29–7.84	0.593
Addiction Clinic Visit (Yes vs. No)	3.15	0.51–19.44	0.204
Surgery History (Yes vs. No)	0.59	0.18–1.94	0.348
Physical Activity—Regular (vs. None)	1.71	0.49–5.97	0.392
Physical Activity—Less Regular (vs. None)	1.24	0.37–4.15	0.719
**Diabetes Mellitus (Yes vs. No)**	**3.59**	**1.01–12.78**	**0.039**
Hypertension (Yes vs. No)	0.93	0.18–4.79	0.933
Blood Transfusion/Donation (Yes vs. No)	0.85	0.21–3.45	0.811
Travel—Africa (vs. No travel)	3.71	0.50–27.54	0.170
Travel—Americas (vs. No travel)	0.12	0.01–1.96	0.128
Travel—Arab Gulf (vs. No travel)	0.33	0.06–1.81	0.161
Travel—Asia (vs. No travel)	1.44	0.25–8.31	0.647
Travel—Europe (vs. No travel)	0.87	0.12–6.31	0.881
Travel—Middle East (vs. No travel)	0.70	0.08–6.13	0.722
**Massage Exposure (Yes vs. No)**	**3.99**	**1.16–13.73**	**0.025**
**Family History of HBV (Yes vs. No)**	**2.55**	**1.10–5.91**	**<0.001**

* Bold values indicate statistical significance (*p* < 0.05). Model diagnostics: Tjur’s pseudo-R^2^ = 0.281.

## Data Availability

The data presented in this study is available on request from the corresponding author. The data is not publicly available due to ethical restrictions and privacy concerns related to sensitive health information.

## Abbreviations

The following abbreviations are used in this manuscript:

HBV	Hepatitis B Virus
HBsAg	Hepatitis B Surface Antigen
GASTAT	General Authority for Statistics
OR	Odds Ratio
SAR	Saudi Riyal
